# Exploring the Dynamics of Immune Checkpoint Inhibitor‐Induced Eosinophilia in Advanced/Metastatic Melanoma: A Comprehensive Retrospective Analysis

**DOI:** 10.1002/cam4.70679

**Published:** 2025-03-27

**Authors:** Panagiotis T. Diamantopoulos, Aikaterini Gkoufa, Amalia Anastasopoulou, Panagiotis Kouzis, Georgios Lyrarakis, Georgios Kyriakakis, Helen Gogas

**Affiliations:** ^1^ First Department of Internal Medicine Laikon General Hospital, National and Kapodistrian University of Athens Athens Greece

**Keywords:** eosinophilia, immune check point inhibitors, immune related adverse events, immunotherapy, melanoma

## Abstract

**Background:**

Immune‐related eosinophilia has emerged as an adverse event associated with immune checkpoint inhibitors (ICIs). Its prevalence, severity, duration, clinical significance, diagnostic approach, and management remain unexplored.

**Methods:**

We conducted a retrospective review of melanoma patient records at a university referral center. Our analysis encompassed the incidence of eosinophilia, baseline disease characteristics, treatment modalities, peak eosinophil counts, associated symptoms, diagnostic procedures, management strategies, disease course, and prognostic implications.

**Results:**

A total of 308 patients were included. Eosinophilia was present in 21.4%, and there was no association with gender, age, histologic type, stage, or BRAF mutation status. The median time interval from treatment initiation to the eosinophilia onset was 56 days, the median eosinophil count at first presentation was 0.70 × 10^9^/L, and the maximum eosinophil count was 1.02 × 10^9^/L. The rate of eosinophilia was significantly higher in patients treated with nivolumab plus bempegaldesleukin (50.0%), followed by nivolumab plus ipilimumab (21.7%). Symptomatic patients and/or patients with hypereosinophilia were assessed for organ involvement and for the identification of the cause of eosinophilia. Patients requiring medical intervention were managed with corticosteroids or antihistamines. Eosinophilia relapsed in 31.8% when rechallenged. While non‐significant, there was a numeric trend for longer overall survival in patients with eosinophilia (42.6 vs. 27.9 months, *p* = 0.178).

**Conclusions:**

This study marks the first comprehensive approach of the relationship between the type of immunotherapy and the incidence of eosinophilia in melanoma patients. It also delves into the patients' baseline characteristics, diagnostic assessment, management, and prognosis, providing useful guidance for physicians treating patients with ICIs.

## 1 Introduction

In recent years, immune checkpoint inhibitors (ICIs), a new class of therapeutic agents, have been introduced in oncology. These drugs act by modulating host immune responses against tumor cells, promoting their elimination, and altering the tumor microenvironment [1, 2]. Soon after ICIs became increasingly used, several immune‐related adverse events (irAEs) emerged, while many studies have evaluated their role as potential biomarkers for disease prognosis and brought strong evidence that their occurrence has been associated with better treatment response [3, 4, 5].

Among hematological irAEs, peripheral eosinophilia has already been described in patients receiving immunotherapy for melanoma and suggested to be related with better clinical response and treatment efficacy [6, 7, 8, 9]. However, a structured investigation algorithm is currently absent from the literature. The available evidence does not report severe, symptomatic eosinophilia requiring discontinuation of ICIs, though safety aspects regarding their administration in cancer patients require further investigation and probably highlight the need for a closer monitoring. The impact of peripheral eosinophilia on cancer treatment decisions (continuation, withholding, permanent discontinuation), as well as on disease prognosis by enhancing antitumor responses, also deserves a deeper insight, with large cohort studies, as eosinophil counts represent an easily available blood test, which may be used as a predictive biomarker.

This study was designed to evaluate and analyze peripheral eosinophilia occurring during the administration of ICIs in melanoma patients and shed light on its significance regarding clinical associations, severity, and impact on patient management, definition of distinct syndromes, as well as the clinical outcome of the disease. In particular, we explore the link between ICIs and eosinophilia, the different types of ICIs responsible for its occurrence, the duration and grade of eosinophilia, the related symptoms or other concomitant irAEs, and provide data on the evaluation of patients with melanoma developing eosinophilia under ICIs, the need for treatment, the treatment choices, as well as melanoma treatment response in this group of patients.

## 2 Methods

We retrospectively collected data from patients with melanoma treated with ICIs in our center in order to analyze the rate of eosinophilia, its clinical features, and its implications in the ICI treatment and prognosis of the patients. Additionally, this manuscript outlines the diagnostic procedures employed for patients who developed eosinophilia during ICI treatment in our institution, along with the corresponding management strategies.

### 2.1 Patients

Adult patients with advanced or metastatic melanoma treated in our institution (a tertiary melanoma reference center) with ICIs from June 2015 until February 2023 were included in the analysis. We recorded and analyzed demographic, baseline clinical, and laboratory data, as well as detailed treatment data for all patients. All ICI‐containing regimens were documented (monotherapy or combination, treatment intension, duration of treatment, and adverse events). Prognostic parameters and survival data were also recorded and analyzed.

### 2.2 Eosinophilia Definitions and Data Collection

Eosinophilia was defined as a peripheral blood eosinophil count equal to or > 0.5 × 10^9^/L, while hypereosinophilia was defined as a peripheral blood eosinophil count > 1.5 × 10^9^/L. According to the Common Terminology Criteria for Adverse Events (CTCAE) v5 classification and severity grading scale for eosinophilia, Grade 1 is defined as an eosinophil count above the upper limit of normal or baseline count, while Grade 3 is assigned when steroids are administered.

We recorded the emergence of eosinophilia and hypereosinophilia during ICI treatment (baseline eosinophil count, time from ICI initiation to eosinophilia development, initial and maximum eosinophil count in patients with eosinophilia, duration of eosinophilia), as well as clinical features accompanying eosinophilia. Patients with preexisting conditions associated with eosinophilia were not excluded from the analysis in order to ensure that the study represents the general population and investigate the potential existence of any association. Moreover, the investigation and management of eosinophilia were also recorded in all patients. Correlations with survival parameters of the cohort with the presence of eosinophilia were also performed. Overall survival (OS) was defined as the time from diagnosis to death from any cause, while progression‐free survival (PFS) as the time period during and after the administration of the ICI associated with eosinophilia. Survival from immunotherapy initiation (OS_I_) was defined as the time from immunotherapy initiation to death from any cause.

### 2.3 Statistical Analysis

All statistical analyses were conducted using IBM SPSS statistics, version 26 (IBM Corporation, North Castle, NY, USA). The Pearson chi‐squared test (or Fisher's exact test when expected values were < 5) was used to test for associations between categorical variables. The Independent‐Samples Mann–Whitney *U* test and the Kruskal–Wallis *H* test were used for testing between not normally distributed continuous variables and a categorical variable with two or more levels respectively. One‐way ANOVA was performed to compare the effect of three or more independent variables to continuous variables. Moreover, The Tukey's post hoc test was used to identify pairs of means that were significantly different. Survival analysis was performed using the Kaplan–Meier method. The level of significance for all statistical tests was set at a probability value of < 5% (two‐sided *p* < 0.005).

The study was approved by the Institutional Review Board of Laikon General Hospital, Athens, Greece.

## 3 Results

### 3.1 Baseline Characteristics of the Patients

At the time of study design, our cohort comprised 308 patients with advanced or metastatic melanoma (stages III–IV using the American Joint Committee on Cancer (AJCC) Staging eighth edition) treated with immunotherapy agent‐containing regimens [10]. The main demographic and clinicopathological characteristics of the patients are shown in Table 1.

**TABLE 1:** Baseline characteristics of the patients.

Characteristics	Result
Number of patients, *N* (%)	308 (100.0)
Caucasian race, *N* (%)	308 (100.0)
Gender (male), *N* (%)	164 (53.2)
Age (years), median (range)	60.9 (21–92)
Histologic type, *N* (%)	
Nodular	79 (25.6)
Superficial spreading	98 (31.8)
Lentigo maligna	13 (4.2)
Acral lentiginous	17 (5.5)
Amelanotic	1 (0.3)
Desmoplastic	1 (0.3)
Spindle cell	2 (0.6)
Not otherwise specified	19 (6.2)
Mucosal	2 (0.6)
Occular	3 (1.0)
Unknown primary	39 (12.7)
Missing data	34 (11.0)
Stage (at diagnosis), *N* (%)	
Ia	7 (2.4)
IIa	30 (10.4)
IIIa	10 (3.5)
Ib	18 (6.3)
IIb	34 (11.0)
IIIb	37 (12.0)
Ic	7 (2.3)
IIc	24 (7.8)
IIIc	77 (25.0)
IIId	5 (2.6)
IV	59 (19.2)
BRAF mutation status, *N* (%) (among 265 tested patients)	
Mutated	122 (46.0)
Wild type	143 (54.0)

### 3.2 Main Eosinophilia Characteristics

Eosinophilia was found in 66 (21.4%) patients, all of whom were under immunotherapy. The baseline eosinophil count of all 66 patients was within normal limits (median 0.26 × 10^9^/L, range 0.03 × 10^9^/L to 0.47 × 10^9^/L). The median eosinophil count at the first emergence of eosinophilia was 0.70 × 10^9^/L (range 0.50–4.53 × 10^9^/L). The presence of eosinophilia was not associated with the baseline characteristics of the patients, namely gender (*p* = 0.487), age (*p* = 0.487), histologic type (*p* = 0.538), stage at diagnosis (*p* = 0.413), or BRAF mutation status (*p* = 0.102).

### 3.3 Association With Immunotherapy Regimens

The immunotherapy regimens administered to the patients during the development of eosinophilia are shown in Table 2. The vast majority of patients (*N* = 57, 86.4%) experienced eosinophilia during first‐line immunotherapy, while seven (10.6%) did not have eosinophilia during their first‐line treatment with ICIs but developed eosinophilia when treated with a subsequent immunotherapy regimen, and two (3.0%) only when treated with a third one. Eosinophilia was identified at a median time of 56 days (range 10–683 days) from treatment initiation.

**TABLE 2:** Regimens administered to the patients (the percentages do not add up to 100, because several patients have been administered more than one regimen).

Immunotherapy regimen, *N* (%)	Result
Nivolumab	142 (46.1)
Pembrolizumab	67 (21.8)
Atezolizumab	9 (2.9)
Ipilimumab	39 (12.7)
Nivolumab with bempegaldesleukin (NKTR‐214)	24 (7.8)
Ipilimumab with nivolumab	59 (19.2)
Atezolizumab with cobimetinib	8 (2.6)
Pembrolizumab with talimogene laherparepvec (T‐VEC)	20 (6.5)
Nivolumab with relatlimab	3 (1.0)
Atezolizumab with vemurafenib and cobimetinib	6 (1.9)
Certalizumab with dabrafenib and trametinib	1 (0.3)
Pembrolizumab with MK1308	8 (2.6)
Interferon alfa	10 (3.2)
Pembrolizumab with tumor infiltrating lymphocytes	1 (0.3)
Pembrolizumab with lenvatinib	1 (0.3)
BRAF/MEK inhibitor	95 (30.8)
Chemotherapy (dacarbazine, paclitaxel)	54 (17.5)

Due to the high variability of the administered treatment regimens, the latter were grouped into eight broader categories. Those included PD1‐inhibitor monotherapy, CTLA4‐inhibitor monotherapy, PD1/CTLA4‐inhibitor combination, PD1/BRAF‐MEK inhibitor combination, PD1‐inhibitor/talimogene laherparepvec (T‐VEC) combination, PD1‐inhibitor/relatlimab combination, PD1‐inhibitor/lenvatinib combination, and PD1‐inhibitor/bempegaldesleukin (NKTR‐214) combination. Moreover, only patients developing eosinophilia during their first immunotherapy‐regimen administration (*N* = 57) were compared to patients not developing eosinophilia under immunotherapy (patients developing eosinophilia during their second or third‐line treatment (*N* = 7, *N* = 2 respectively) were excluded from the analysis).

A one‐way ANOVA revealed that there was a statistically significant difference (*p* < 0.0001) in the eosinophil count between at least two of the above‐mentioned treatment regimens. Tukey's test for multiple comparisons found that the mean value of the eosinophil count was significantly higher in patients treated with the PD1‐inhibitor/NKTR‐214 combination compared to those treated with several other immunotherapy regimens, that is, PD1‐inhibitor monotherapy (*p* < 0.0001; 95% CI, 140.0–720.8), PD1/CTLA4‐inhibitor combination (*p* = 0.04; 95% CI, 11.1–862.3), PD1‐inhibitor with BRAF/MEK inhibitor (*p* = 0.044; 95% CI, 6.1–778.5), and PD1‐inhibitor with T‐VEC (*p* = 0.044, 95% CI, 6.3–938.7).

The eosinophil count at the first presentation of eosinophilia did not differ among the immunotherapy regimens (*p* = 0.561). The same applied to the duration of eosinophilia (*p* = 0.253). Moreover, the maximum eosinophil count observed during the follow‐up of patients for eosinophilia did not differ among patients treated with different immunotherapy regimens (*p* = 0.482).

### 3.4 Duration and Severity of Eosinophilia

The median duration of eosinophilia at its first emergence was 57 (2–843) days, and the median maximum eosinophil count during the course of eosinophilia was 1.02 × 10^9^/L (0.5–13.22 × 10^9^/L). Hypereosinophilia was present in 3/66 (4.5%) patients at the first development of eosinophilia, and in 21/66 (31.9%) during follow‐up. Hypereosinophilia was more common in patients treated with the combination of nivolumab and NKTR‐214 than in patients treated with other immunotherapy regimens (*p* = 0.017). Moreover, patients with hypereosinophilia had a longer median duration of eosinophilia (80 vs. 28 days for patients with lower eosinophil counts, *p* = 0.005).

### 3.5 Clinical Characteristics and Diagnostic Evaluation of Eosinophilia

Most cases of eosinophilia were asymptomatic, with the increase in eosinophils primarily identified as a laboratory finding. Only 15 (22.7%) patients presented with other symptoms at the development of eosinophilia, which included pruritus, rash, and arthralgia, while fever, diarrhea, cough, flu‐like symptoms, and angioneurotic edema were rare. In those cases, eosinophilia along with the accompanying symptoms were considered as part of the same syndrome, and not as different AEs. No cases of cardiac or neurologic involvement were noted (Table 3).

**TABLE 3:** Characteristics of patients with eosinophilia. Comparisons with patients without eosinophilia.

Characteristic	Result
Patients with eosinophilia, *N* (%)	61 (24.5)
Under immunotherapy at the development of eosinophilia, *N* (%)	61 (100.0)
Immunotherapy duration at the development of eosinophilia (days), median (range)	55 (10–534)
Eosinophilia percentage per treatment regimen, *N* (%)	
Nivolumab	17/103 (16.5)
Pembrolizumab	6/54 (11.1)
Atezolizumab	1/9 (11.1)
Ipilimumab	5/37 (13.5)
Nivolumab with bempegaldesleukin (NKTR‐214)	12/24 (50.0)
Ipilimumab with nivolumab	10/46 (21.7)
Atezolizumab with cobimetinib	2/8 (25.0)
Pembrolizumab with talimogene laherparepvec (T‐VEC)	3/20 (15.0)
Nivolumab with relatlimab	0/3 (0.0)
Atezolizumab with vemurafenib and cobimetinib	3/6 (50.0)
Certalizumab with dabrafenib and trametinib	1/1 (100.0)
Pembrolizumab with MK1308	1/8 (12.5)
Interferon alfa	0/10 (0.0)
Pembrolizumab with tumor infiltrating lymphocytes	0/1 (0.0)
Eosinophil count at first presentation of eosinophilia (×10^9^/L), median (range)	0.70 (0.50–4.53)
Maximum eosinophil count (×10^9^/L), median (range)	1.07 (0.50–13.22)
Symptoms at the development of eosinophilia, *N* (%)	
Pruritis	6 (9.8)
Rash	6 (9.8)
Arthralgia	3 (4.9)
F ever	1 (1.6)
Cough	1 (1.6)
Angioneurotic eodema	1 (1.6)
Flu‐like symptoms	1 (1.6)

Regarding the medical history and comorbidities of the patients, eosinophilia was not associated with the presence of comorbidities possibly associated with a higher eosinophil count, such as autoimmune diseases, hypothyroidism, asthma/rhinitis, dermatoses, or myeloproliferative neoplasms.

The diagnostic evaluation of patients presenting with eosinophilia depended on the eosinophil count and the presence of symptoms. All patients were evaluated for constitutional symptoms, cutaneous, cardiac, gastrointestinal, respiratory, or neurological symptoms, while a review of the patient's medications and diet was also performed. A thorough physical examination seeking evidence of organ involvement and possible causes of eosinophilia was carried out. None of the patients was acutely ill requiring urgent evaluation and management. Asymptomatic patients without hypereosinophilia or clinical findings were offered a repeat complete blood count in 1 or 2 weeks and before their next treatment cycle. If eosinophilia persisted, further evaluation was performed. Symptomatic patients, or patients with hypereosinophilia, were assessed for organ involvement depending on the symptoms, and for the identification of the cause of eosinophilia. This group of patients was evaluated with a complete blood count and blood smear, a biochemistry profile including lactate dehydrogenase and uric acid, cardiac troponin, a chest X‐ray, and a heart ultrasound. Further evaluation was performed according to the clinical scenario and the findings of the evaluation (e.g., stool test for ova and parasites, in patients with diarrhea or recent travel, IgE and tryptase for selected patients). One patient with dermatologic manifestations was ultimately diagnosed with bullous pemphigoid. None of the patients was found to have an underlying cause of eosinophilia other than treatment with immunotherapy.

### 3.6 Management of Eosinophilia

The therapeutic approach depended on the presence of symptoms and/or clinical/laboratory findings, the eosinophil count, and whether a definitive diagnosis was reached. Symptomatic patients, without organ damage, were managed with corticosteroids, while asymptomatic patients were most of the time left untreated except in cases of hypereosinophilia. In brief, 22 (33.3%) patients received systematic treatment, 20 of them with oral steroids and seven with antihistamine agents. Topical steroids were first prescribed in six patients presented with rash, without improvement. In steroid‐treated patients, oral methylprednisolone or prednisolone was used. The used doses ranged from 0.1 to 1 mg/kg of prednisolone or equivalent. Treatment with steroids was effective in all cases in terms of symptom control and relief and eosinophilia remission, permitting fast tapering after the first week. Thirteen patients needed a maintenance dose of around 10 mg of prednisolone or equivalent. Immunotherapy was temporarily interrupted in 21/66 (31.8%) patients. In the vast majority of them (20/21, 95.2%) eosinophilia relapsed when they were rechallenged with the same or a different immunotherapy regimen. Eosinophilia relapse was managed in all cases with low‐dose prednisolone or equivalent. It was not feasible to demonstrate a definitive trend in eosinophil levels due to variations in patient management. Some continued immunotherapy, others received systemic steroids, and in some cases, immunotherapy was withheld.

### 3.7 Prognostic Correlations

**FIGURE 1:**
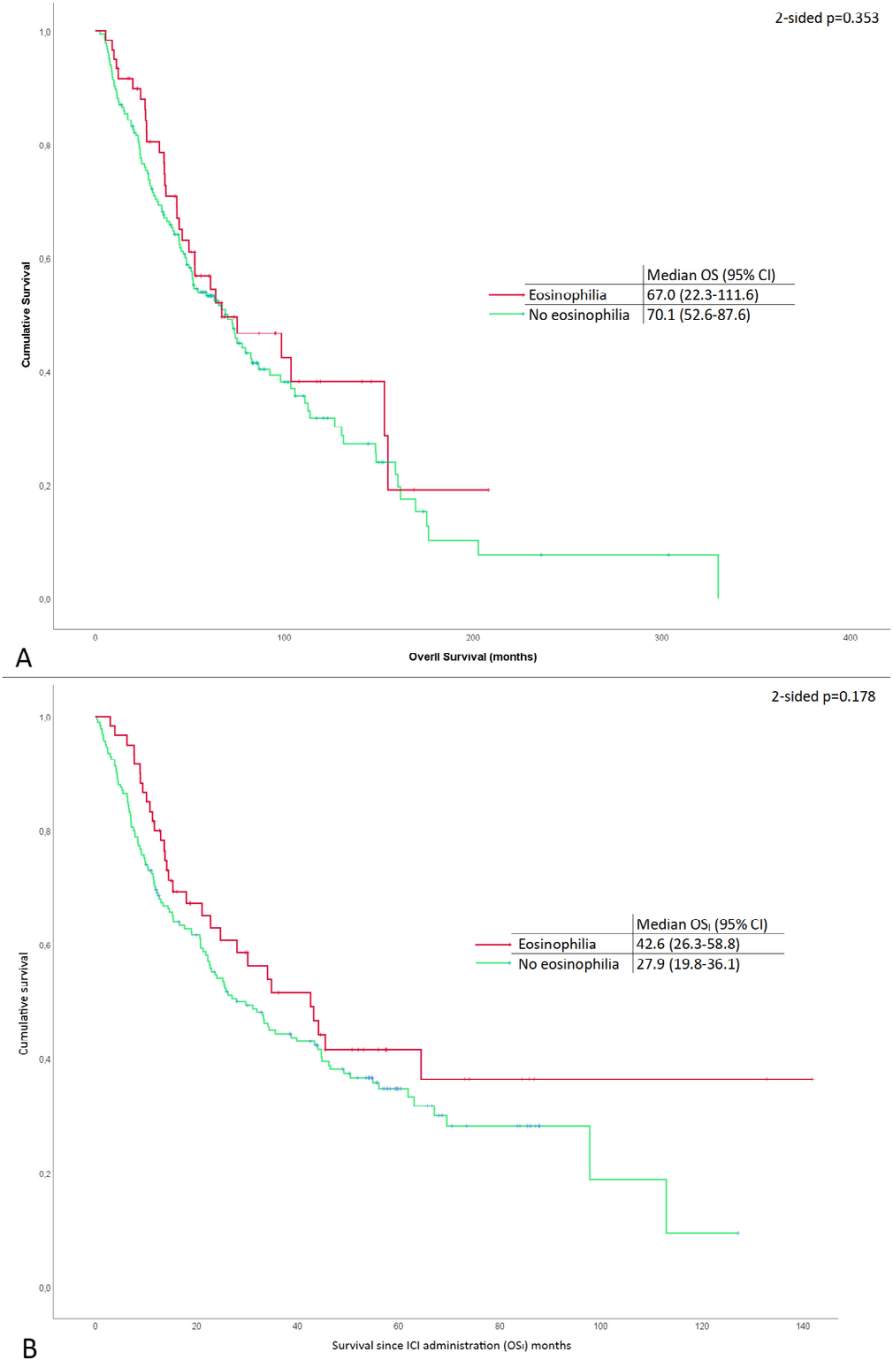
Kaplan–Meier overall survival analysis (A) and survival since ICI administration (B) for patients with and without eosinophilia.

Eosinophilia was not correlated with any of the survival parameters that were analyzed. In detail, there was no difference in OS in patients with eosinophilia and without eosinophilia (67.0 months; 95% CI, 22.3–111.6 vs. 70.1 months; [95% CI, 52.6–87.6], 2‐sided *p*, 0.353). Although not statistically significant, there was a numerical trend suggesting higher overall survival (OSI) in patients with eosinophilia (42.6 months [95% CI, 26.3–58.8]) compared to those without eosinophilia (27.9 months [95% CI, 19.8–36.1]), with a two‐sided *p*‐value of 0.178. The corresponding Kaplan–Meier curves are shown in Figure 1. Moreover, there were no differences between patients with and without eosinophilia in terms of OS or OS_I_ when analyzing the survival of patients treated in the adjuvant or the metastatic setting.

There was no observed correlation between eosinophilia and treatment response. Among patients with metastatic disease, the incidence of eosinophilia did not differ between those achieving objective responses (CR and PR) and non‐responders (SD/PD) (*p* = 0.545). Similarly, no significant difference was noted when comparing patients with PD to all others (*p* = 0.328). Furthermore, progression‐free survival (PFS) in metastatic patients did not significantly differ based on the presence or absence of eosinophilia (7.8 months in patients with eosinophilia vs. 5.9 months in those without, *p* = 0.435). Finally, at the time of immunotherapy administration, 58 patients (18.8%) had brain metastases. In those patients, the presence of eosinophilia was not correlated with differences in OS or OS_I_.

## 4 Discussion

Peripheral eosinophilia emerging during treatment with ICIs in melanoma patients represents a not well‐defined adverse event, with unknown prognostic implications, that may impact therapeutic decisions. Although eosinophilia can sometimes be attributed to the underlying malignancy, independently of the administered antitumor agents [7], emerging therapeutic modalities, including ICIs, which display high efficacy in the management of cancer, have been correlated with hematological adverse events [11]. However, due to their rarity, incidence, clinical course, and prognostic significance, they require further investigation. Several studies, trying to identify useful biomarkers associated with melanoma response to ICI administration, have demonstrated that an increase of eosinophils during immunotherapy in those patients has been associated with a higher OS rate [8, 12, 13]. However, there is a trend of solely investigating eosinophilia as a prognostic biomarker, overlooking other potential clinical implications, leading to a scarcity of data regarding associations of eosinophilia with the immunotherapy regimen, as well as regarding other characteristics of eosinophilia such as duration, severity, and diagnostic evaluation, all of which have been rarely explored.

This is the first study analyzing peripheral eosinophilia as an irAE in patients with metastatic melanoma who received different ICI‐containing regimens, uncovering the incidence rates of eosinophilia with regards to the characteristics of patients, while this study represents the first effort for the establishment of a standardized diagnostic approach and management of peripheral eosinophilia induced by ICIs in melanoma patients. As indicated in the literature, the administration of ICIs has been associated with peripheral eosinophilia [7, 8, 14, 15]. Overall, our findings corroborate existing results. Furthermore, all patients in our cohort had baseline eosinophil counts within normal limits, and the increase in eosinophil count was reliably attributed to the administered regimens. When compared with a previous observational study, which investigated patients with different malignancies [16], our study reported a higher incidence of eosinophilia in melanoma patients receiving immunotherapy (2.8% vs. 21.4%). However, Tessier et al. had excluded patients who received a combination of ICIs [16]. Interestingly, this is the first report including exclusively patients suffering from advanced or metastatic melanoma and receiving monotherapy or combinations of ICIs. Of note, those treated simultaneously with nivolumab and bempegaldesleukin (NKTR‐214) or nivolumab and ipilimumab experienced eosinophilia more frequently compared with patients treated with a single agent, while the combination of nivolumab and bempegaldesleukin (NKTR‐214) was more commonly associated with hypereosinophilia, as well as with longer duration of eosinophilia. This observation might not come as a surprise to clinicians, given that bempegaldesleukin functions as a CD122‐preferential interleukin‐2 pathway agonist. According to literature data, IL‐2 may stimulate IL‐5–producing group 2 innate lymphoid cells, which can, in turn, lead to eosinophilia [17]. Interestingly, based on published literature, administration of bempegaldesleukin in combination with nivolumab increased toxicity and provided no further clinical benefit compared with nivolumab monotherapy, and as of now, this agent has not received approval for any type of cancer [18]. Although there is a detailed approach for patients with common irAEs affecting mainly the skin, gastrointestinal tract, and endocrine organs, as well as for rare but serious neurological complications [19, 20], published data regarding eosinophilia in the context of ICI administration is missing. Due to this dearth of literature data, a reasonable concern for clinicians confronting patients under ICI therapy presenting with eosinophilia is whether the increase of eosinophil count refers to a simple laboratory abnormality or is accompanied by organ involvement. In a small cohort study monitoring 37 cancer patients with eosinophilia induced by ICIs, 57% of them presented with organ involvement, while no deaths were reported due to eosinophilia [9]. Moreover, 70% of patients discontinued immunotherapy after this irAE. In our cohort, eosinophilia most commonly occurred asymptomatically and was typically documented in routine laboratory monitoring. Accompanying symptoms in 22.7% of patients were only mild, and no serious events, such as cardiac or neurologic involvement, were documented. Based on these data and understanding that hypereosinophilia could potentially affect target organs through infiltration, frequent laboratory monitoring, together with a thorough clinical evaluation of this group of patients, is advisable, since guidelines for the diagnostic approach and management of this entity are still lacking.

**FIGURE 2:**
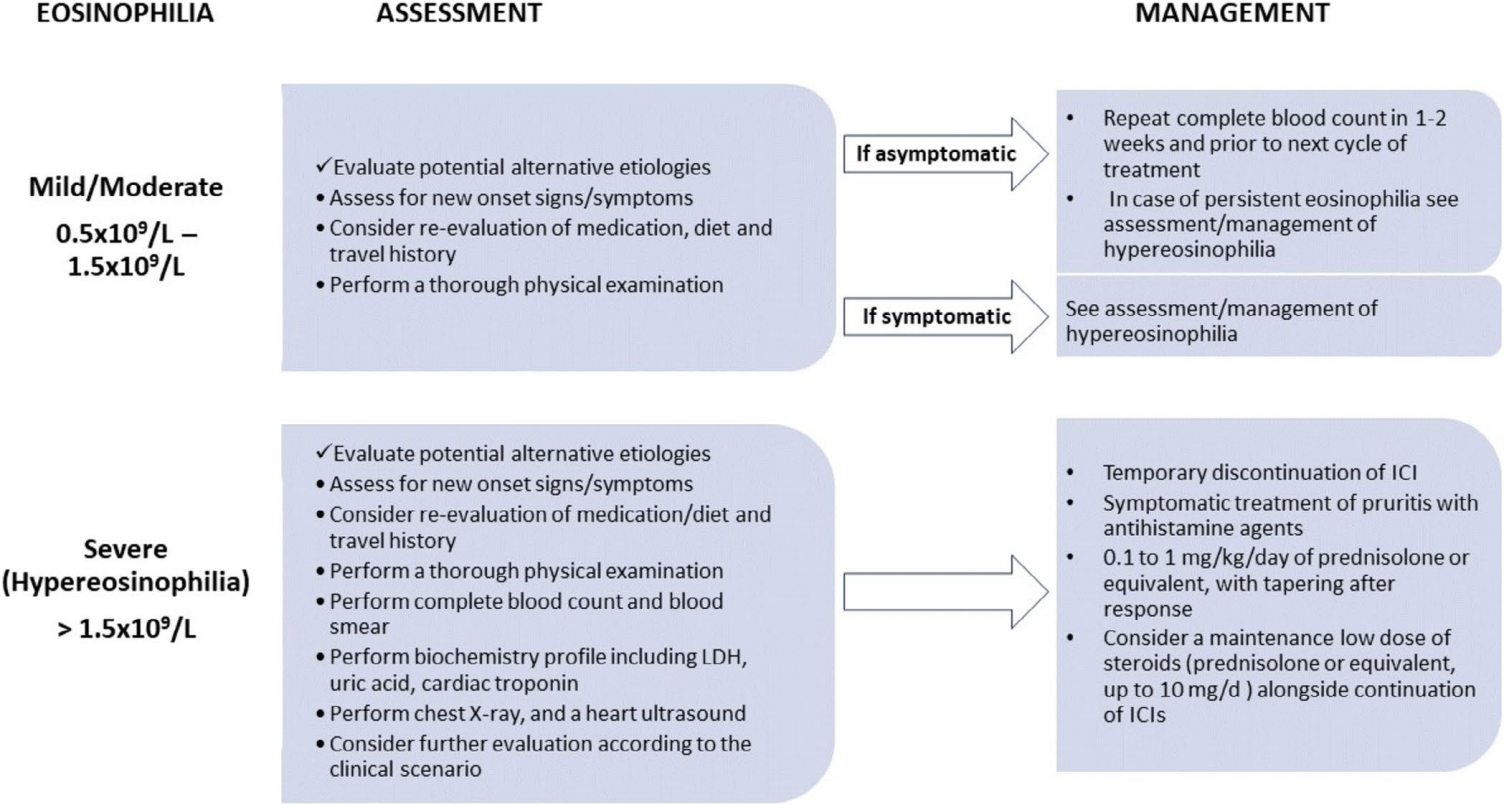
A proposed algorithm for the diagnostic and therapeutic approach of immunotherapy‐induced peripheral eosinophilia.

Based on the World Health Organization (WHO) guidelines regarding the approach of patients with eosinophilia [21], when documented, clinicians should first review patients' medical history in order to identify possible secondary conditions, such as comorbidities, infections (mainly parasitic), atopic diseases, or substance use which could be related to eosinophilia and treat as needed. A detailed clinical assessment plays a pivotal role for the early detection of organ involvement. If eosinophilia represents just a laboratory abnormality, patients should be followed up, together with a repeated eosinophil count, before their next cycle of ICI administration. However, in the case of hypereosinophilia and/or symptomatic patients, further laboratory and imaging tests should be performed, as organ damage may occur irrespective of the underlying cause or the eosinophilia level and should be mainly guided by clinical findings, aiming for a timely recognition of severe organ damage (e.g., heart, lung, nervous system). Re‐evaluation should be arranged earlier and before the next immunotherapy treatment cycle. Figure 2 illustrates a useful algorithm for the diagnostic and therapeutic approach of immunotherapy‐induced peripheral eosinophilia.

In this study, the majority of patients with hypereosinophilia did not present with organ involvement. Only one patient developed bullous pemphigoid after nivolumab administration and was successfully treated with oral prednisolone [22]. It is noteworthy that due to frequent cutaneous toxicities associated with ICIs, a skin biopsy specimen should be obtained for histopathological evaluation and direct immunofluorescence, in addition to serologic evaluation [22], especially in patients presenting with pruritus and papular lesions, which progress to bullae. Similarly, in the cohort of Tessier et al., patients with immune‐related eosinophilia were asymptomatic [16]. On the other hand, Scanvion et al. described cases with severe eosinophilic‐related organ damage after ICI administration [9]. All these data underline the need for a careful approach for symptomatic and/or asymptomatic patients with hypereosinophilia, despite the benign course for the majority of them, while the prolonged duration of eosinophilia reported in our cohort probably indicates the need for a long follow‐up of patients.

Regarding management, as already mentioned, hypereosinophilia and/or patients with severe symptoms or organ damage warrant intervention. Therapeutic modalities may include frequent follow‐up, temporary ICI discontinuation, and administration of antihistamine agents and corticosteroids, while clinicians should deal with the challenge of selecting the optimal management. Patients with eosinophilia and concurrent troublesome symptoms, like pruritus, could easily be managed with antihistamines, while for patients with persistent symptoms or for those who do not achieve an adequate response, a short course of low‐dose corticosteroids with temporary treatment discontinuation has been proven to be effective (Figure 2). No other immunomodulators were required to control symptomatic patients or hypereosinophilia in our cohort, compared with other refractory irAEs, which may require an escalating strategy with more intense immunosuppressive therapy. Importantly, as a complete recovery and adequate management were achieved in all patients without the need for permanent treatment withdrawal, even for cases where relapses occurred after ICI rechallenge, the decision for permanent treatment cessation should be well‐balanced and should be avoided in the vast majority of cases, especially considering that melanoma still represents an aggressive malignancy with metastatic behavior and high mortality rates. However, limited literature exists documenting cases where discontinuation of ICIs and steroid administration were insufficient to resolve hypereosinophilia [23]. In such instances, physicians successfully employed an IL‐5 blocking agent as an alternative therapeutic approach [23].

Given the paucity of data and the lack of clarity on whether ICI‐induced eosinophilia may lead to irreversible complications such as fibrosis, discontinuation of ICIs remains an unsupported decision. Prioritizing patient safety, we opted to maintain a low dose of steroids in melanoma patients exhibiting hypereosinophilia after ICIs, thereby enabling the continued administration of these agents, a decision with a substantial impact on patient prognosis. In different scenarios, we might have considered leaving asymptomatic hypereosinophilia untreated, with regular monitoring.

Regarding the prognostic implications of eosinophilia, and driven from the existing literature data, which suggest that the occurrence of irAEs is likely linked to improved response and survival rates [24, 25] we further analyzed survival rates in patients who developed eosinophilia following ICIs administration. Our study did not identify a significant association between its occurrence and overall survival, despite an observed trend toward a higher median OS_I_ in patients with eosinophilia.

To date, this manuscript represents the largest study investigating the occurrence of eosinophilia after ICI administration, either as monotherapy or in combination regimens, within a homogeneous population, as it exclusively comprises patients with advanced/metastatic melanoma. It also provides a comprehensive description of the diagnostic evaluation and therapeutic interventions that should be employed for these patients, to provide useful information and assist other clinicians in effectively addressing this immune‐mediated complication. However, a significant limitation pertains to the study's retrospective, single‐center design. Finally, further studies could probably investigate the correlation between peripheral and tumor‐associated tissue eosinophilia, as there is an emerging perspective that eosinophilia in the cancer microenvironment might be linked to more favorable clinical outcomes [26].

## 5 Conclusions

As the use of ICIs increases, the incidence of irAEs also rises, necessitating vigilance among clinicians to promptly identify and assess their clinical significance and impact. While immune‐related eosinophilia is a recognized adverse event following ICIs administration, its diagnostic approach, follow‐up strategy, and management have not been clearly defined until now. This large, homogeneous cohort study establishes the foundation for a well‐organized approach and management of immune‐related eosinophilia, addressing the existing gap in knowledge.

## Author Contributions

**Panagiotis T. Diamantopoulos:** conceptualization (equal), formal analysis (equal), writing – original draft (equal). **Aikaterini Gkoufa:** writing – original draft (equal). **Amalia Anastasopoulou:** writing – original draft (equal). **Panagiotis Kouzis:** writing – review and editing (equal). **Georgios Lyrarakis:** validation (equal). **Georgios Kyriakakis:** validation (equal). **Helen Gogas:** conceptualization (equal).

## Ethics Statement

The study was approved by the Institutional Review Board of the participating center (Laikon General Hospital, Athens, Greece, IRB protocol number 67/25.01.21).

## Consent

For anonymous publication of retrospective data acquired during routine clinical care, individual informed consents were not obtained.

## Conflicts of Interest

The authors declare no conflicts of interest.

## Data Availability

All data are available upon reasonable request.
